# *Plasmodium falciparum *gametocyte dynamics in areas of different malaria endemicity

**DOI:** 10.1186/1475-2875-7-249

**Published:** 2008-12-03

**Authors:** Kasia Stepniewska, Ric N Price, Colin J Sutherland, Chris J Drakeley, Lorenz von Seidlein, Francois Nosten, Nicholas J White

**Affiliations:** 1 Mahidol-Oxford Tropical Medicine Research Unit, Faculty of Tropical Medicine, Mahidol University, 420/6 Rajvithi Road, Bangkok 10400, Thailand; 2 Centre for Clinical Vaccinology and Tropical Medicine, Churchill Hospital, Oxford, UK; 3 International Health Division, Menzies School of Health Research and Charles Darwin University, Darwin, NT, Australia; 4 Department of Infectious & Tropical Diseases, London School of Hygiene and Tropical Medicine, UK; 5 Joint Malaria Programme, Tanga, Tanzania; 6 Shoklo Malaria Research Unit, Tak, Thailand

## Abstract

**Background:**

The aim of this study was to identify and compare factors associated with *Plasmodium falciparum *gametocyte carriage in three regions of differing malaria endemicity.

**Methods:**

Retrospective data from Thailand, The Gambia and Tanzania were used. The data came from large prospective field-based clinical trials, which investigated gametocyte carriage after different anti-malarial drug treatments.

**Results:**

Gametocytaemia was detected during the observation period in 12% of patients (931 out of 7548) in Thailand, 34% (683 out of 2020) in The Gambia, and 31% (430 out of 1400) in Tanzania (p < 0.001). Approximately one third (33%, 680/2044) of the patients with gametocytaemia during the observation period, already had patent gametocytaemia at enrolment (day 0 or day 1): 35% (318/931) in Thailand, 37% (250/683) in The Gambia, 26% (112/430) in Tanzania. Maximum gametocytaemia was usually observed on or before the seventh day after starting treatment (93% in Thailand, 70% in Tanzania and 78% in The Gambia). Lowest gametocyte carriage rates were observed following treatment with artemisinin derivatives, while sulphadoxine-pyrimethamine (SP) was associated with significantly greater development of gametocytaemia than other drug treatments (p < 0.001). The duration of gametocyte carriage was shorter in Thailand by 86% and Tanzania by 65% than in The Gambia. Gametocyte carriage was 27% longer among people presenting with anaemia, and was shorter in duration among patients who received artemisinin derivatives, by 27% in Thailand and by 71% in Tanzania and The Gambia.

**Conclusion:**

This study confirms the independent association of gametocytaemia with anaemia, and the significantly lower prevalence and duration of gametocyte carriage following treatment with an artemisinin derivative. The large differences in gametocyte carriage rates between regions with different levels of malaria transmission suggest that drug interventions to prevent transmission will have different effects in different places.

## Background

Malaria control rests traditionally on two strategies; vector control (reducing the numbers of anopheline vectors and reducing the probability of being bitten), and drug treatment. Effective anti-malarials reduce morbidity, prevent mortality, and reduce asexual parasite biomass. This, in turn, reduces the numbers of sexual (transmissible) parasites (gametocytes). In *Plasmodium falciparum *infections, it remains unclear whether gametocyte production is programmed early on after hepatic schizogony or is a response to stimuli acting upon the parasite population. The proportion of parasites committed to sexual stage development may change during the course of an infection. The developing sexual stages (stages I to IV) remain sequestered in the microvasculature for approximately 10 days before appearing as morphologically distinct male and female stage V gametocytes in the peripheral blood. One male (containing eight microgametes) and one female (macro-gamete) are required per mosquito blood meal (approx 2 μL) for infection to occur. Thus gametocyte densities of 1 per μL are theoretically sufficient to infect mosquitoes, a density beneath the limit of detection for most routine microscopy. This explains malaria transmission from subjects without apparent gametocytaemia.

In areas of low or unstable transmission most malaria infections are transmitted by people who are ill, or recovering from symptomatic malaria. In such areas asymptomatic infections are unusual, so treatment-seeking behaviour and the pharmacokinetic and pharmacodynamic properties of the anti-malarial drugs used are important determinants of transmission. But low transmission settings often contain small foci of higher transmission and the few asymptomatic individuals in these areas are important in sustaining malaria through the dry season. In higher transmission settings the situation is more complex. Anti-disease controlling immunity is acquired which results in an increasing proportion of infections stabilizing at relatively high parasite densities. These may be either asymptomatic or tolerated in older patients who are less likely to seek treatment. These infections may still be transmissible [1]. In addition to immunity against asexual stages there is the development of a specific immunity against sexual stage parasites which can further reduce the transmission probability per infection [2,3]. Several investigators have assessed the contributions of different age and patient groups to overall malaria transmission in endemic areas by direct measurement of infectivity to vector mosquitoes. These studies, which have been conducted in different geographic regions with differing patterns of malaria epidemiology, emphasize the importance of older often asymptomatic individuals in sustaining transmission of both falciparum and vivax malaria despite usually lower parasite densities [4-10].

Recrudescence (treatment failure), and the subsequent extended duration of infection is an important source of transmission particularly of drug-resistant parasite genotypes [11]. The factors associated with gametocytogenesis include the parasite density itself [12,13], anaemia [14], the duration of infection, stresses on the parasite population such as host immunity (related to age) or anti-malarial treatment, and the stage specificity of the anti-malarial drugs used. The relationship between gametocyte density in blood and the transmission probability is generally sigmoid [15-17], although it varies between individuals and is affected by the factors described above [18,19].

The aim of this study was to identify factors associated with gametocyte carriage at different levels of malaria transmission and thus clinical epidemiology. Retrospective data from study sites in three different endemic areas were used. The data come from large prospective field-based clinical trials which investigated gametocyte carriage after different anti-malarial drug treatments.

## Methods

### Description of study sites

#### Tanzania

The Tanzanian studies were carried out near Ifakara, in the Kilombero district in the southeast of the country between 1997 and 1999. At the time falciparum malaria was the foremost health problem in the district. In a one-year period, 37% of children (aged 0–15) admitted to hospital in Ifakara were diagnosed as having malaria with a case fatality rate of 3% [20]. Most of these were infants. At the time these data were collected, the parasite prevalence in rural areas was approximately 70%, rising to 90% in children under five years, and remained high all year-round. Nearly all these cases were *P. falciparum*, although *Plasmodium malariae *and *Plasmodium ovale *infections also occurred occasionally. Entomological inoculation rates (EIRs) were between 200 and 300 infective bites per person per year, with no seasonal variation. In this region, there were a large number of government dispensaries and chloroquine was widely available in local shops. This study on gametocyte dynamics was part of a larger study investigating natural transmission-blocking immunity in this area of intense perennial transmission [21].

#### The Gambia

The Gambian study catchment area was roughly 275 km^2^, located at two sites, one to the east and the other to the west of Farafenni, a rural town 170 km to the east of the Atlantic coast. During cross-sectional malaria surveys, between 7% to 9% of children under five years of age were found to be parasitaemic. The overall the malaria attributable mortality in this region in the 1980s was estimated to be 6.3 per 1,000 in children under the age of one year and 10.7 per 1000 in children aged 1–4 years [22]. Transmission was highly seasonal following the rains in July to September with an EIR of approximately 4 [23] with a peak of clinical attacks at the end of the wet season. Symptomatic malaria was rare between February and July. Data used in our study comes from three studies [16,24,25].

#### Thailand

The studies in Thailand were carried out in camps for displaced persons of the Karen ethnic minority on the western border of Thailand. Transmission of malaria was unstable and seasonal, occurring through out the year with peaks in May-July and December-January [26]. The estimated EIR and corresponding incidence of malaria was low (approximately 0.5 to 1.5 cases/person/year) with prevalence rates of 1–4% for *P. falciparum *[26]. Overall, *P. falciparum *accounted for 37% of malaria infections, with the remainder due to *Plasmodium vivax*. All *P. falciparum *infections and approximately 90% of *P. vivax *infections were symptomatic, but the case fatality rate was low (~0.1%). Severe disease was more common in young children and pregnant women [27]. Between 1989 and 2000, more than 10,000 people were enrolled in more than 25 drug studies. The data come from an amalgamation of 18 studies (mostly comparative trials, but some simply monitoring therapy) conducted between 1990 and 1996 [28,29]. Data from the artemether-lumefantrine studies were gathered between 1996 and 1999 [30,31].

### Design of studies

In all studies symptomatic patients attending outpatient clinics were considered for enrolment. In Thailand, patients of all ages were enrolled providing that they weighed more than five kilograms, whereas in Tanzania, all patients were older than one year (range 1–67 years). In The Gambia all patients were under ten years of age, weighed more than five kilograms and had a parasitaemia greater than 500 per microlitre in the presence of a febrile illness. Fully informed consent was obtained from all patients or their parents or guardians. Pregnant women and patients with severe disease were excluded at all three sites.

Patients were monitored following drug treatment in Thailand weekly until day 28 in early studies, and until day 63 in later studies. In Tanzania post-treatment follow up was weekly until day 28, while in The Gambia follow up was on day 7, day 14 and day 28.

### Parasite counting

Parasite counts were calculated from Giemsa-stained thick and thin blood films. In Thailand, parasitaemia was expressed as the number of parasitized erythrocytes per 1,000 red cells or the number of parasites seen on the thick film per 500 white blood cells; in The Gambia and in Tanzania it was expressed per 200 white blood cells. Gametocyte density was determined on the basis on the number of gametocytes per 500 white blood cells in Thailand and Tanzania, and per 1,000 white cells in The Gambia. In Tanzania slides were declared negative if no parasite was seen in 100 high power fields.

### Description of the data

In all studies patient characteristics were recorded at enrolment including: age, sex, haematocrit, temperature, parasitaemia, length of the illness before enrolment, size of liver, size of spleen and the patient's symptoms. The size of the liver, spleen, the haematocrit and parasitaemia were also measured at the follow-up examinations. In majority of patients in Tanzania parasitaemia was only recorded as a categorical variable with five levels: < 800/μL, 800–1,599/μL, 1,600–3,999/μL, 4,000–19,999/μL and ≥ 20,000/μL. Patients with no recorded parasitaemia on enrolment were excluded from the analyses.

There was a considerable variability in malaria treatments between the studies. In Thailand chloroquine and sulphadoxine-pyrimethamine were not evaluated because of the high level resistance. Artemisinin derivatives either alone or in combination were the only drugs evaluated at all three locations. Eight treatment groups were defined (Table 1).

**Table 1:** Malaria treatments evaluated and their estimated efficacy

**Treatment Group**	**Failure Rate (95% CI)^1 ^(%)** **Number of patients treated**		
	
	**Thailand**	**The Gambia**	**Tanzania**
**Artemisinin derivatives ± other drugs (A)**	15 (14–17) 5324	9 (7–12) 1207	27 (20–36) 130

**Antipyretics only (B)**			33 (13–70) 32

**Chloroquine (C)**		42 (31–55) 120	62 (58–66) 1115

**Halofantrine (D)**	14 (11–18) 459		

**Mefloquine only (E)**	32 (27–37) 1423		

**Quinine regimens (F)**	22 (17–28) 311		75 (31–93) 8

**Sulfadoxine-Pyrimethamine (G)**		8 (5–11) 693	46 (29–67) 70

**Sulfadoxine-Pyrimethamine + Chloroquine (H)**			50 (21–87) 13

^1 ^defined as recurrent parasitaemia within 42 days for Artemisinin derivatives (A) and Mefloquine (E) and 28 days for other treatments; estimated using Kaplan-Meier method.

Four principal outcomes were evaluated:

(i) presence of gametocytes on enrolment (binary);

(ii) appearance of gametocytes after enrolment up to day 14 (binary);

(iii) length of gametocyte carriage within 28 days follow-up (continuous);

(iv) level of maximum gametocytaemia (continuous).

Presence of gametocytes on enrolment was defined as a gametocytaemia detected by microscopy on day 0. Only patients with a recorded count available on day 0 were included in the analysis of this endpoint. Appearance of gametocytes after enrolment up to day 14 was defined in patients with no detectable gametocytaemia on enrolment and, for the purpose of comparison between sites, was assessed only from patients with blood slide assessments on days 7 and 14. For the analysis of factors associated with gametocytaemia within each site, in addition to mandatory counts on days 7 and 14, counts between days 1 and 6, if available, were used to evaluate carriage. Thus for this analysis, if there was a positive count at anytime in the first two weeks, the patient was classified as gametocytaemic regardless of availability of counts on day 7 or 14.

The duration of gametocyte carriage was defined as the time from the first recording of gametocytaemia until half way between the last positive record and the subsequent negative record. For patients with missing records after a positive record of gametocytaemia, half way between the last positive record and the next scheduled assessment (7, 14, 21 or 28 days) was used to calculate duration of carriage, and these observations were treated as censored. For patients with intermittent gametocytaemia, duration of carriage was defined as time from the first positive count to the last positive count recorded. Missing assessments between positive counts were treated as positive. Maximum gametocytaemia was defined as the maximum density of gametocytaemia recorded over the whole follow-up period.

**Figure 1:**
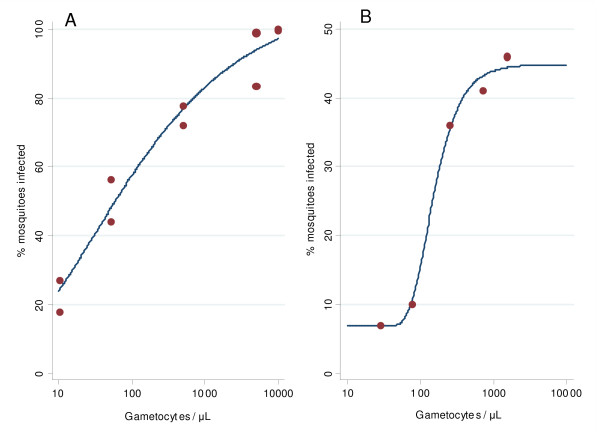
Relationship between infectivity and gametocytaemia level: (A) Jeffery-Eyles (B) Drakeley et al.

Infectivity was not assessed in these studies. Whilst accepting that the relationship between infectivity and parasite densities is dependent on many factors and varies considerably between individuals, there is nevertheless a quantitative relationship between the two. To illustrate this, gametocytaemia-infectivity relationships from two data sets were used to construct putative infectivity. For each patient gametocyte densities on days 0, 7, 14, 28 were used to interpolate (using the straight line interpolation between two subsequent points) levels for all days between day 0 and day 28. These levels were then converted into the probabilities p_i _of infecting a feeding anopheline mosquito on the *i*-th day using a sigmoid relationship (Figure 1) obtained from fitting a non-symmetric Gompertz model to the estimated proportion of infected mosquitoes:

Method A: 1.08 exp(- exp(-0.86*(log_10_(gametocyte count) * – 1.48)))

This is a data set derived from non-immune volunteers [15].

Method B: 0.07 + 0.38 exp(-exp(-4.09 *(log_10_(gametocyte count) – 2.09)))

This is a data set derived from semi-immune children in The Gambia [16].

The model fits were satisfactory (both R^2 ^≥ 0.99) with the estimated values between the observed values close to linear interpolation. The same relationship was used for all treatments.

As a measure reflecting overall infectivity of an individual, the area under the curve (AUIC) of the probability of infecting a biting female anopheline mosquito and time was estimated [17] and expressed in infectivity days. This was calculated using trapezoid rule as any short segment of the infectivity curve could be approximated by a straight line. This measure reflects the potential gametocyte exposure to feeding anopheline mosquitoes, but does not take into account any individual drug effect on infectivity which is independent of gametocyte density (such as sporontocidal activity). As a measure of overall infectivity of a population the individual AUICs from all patients (carrying gametocytes or not) were added up and presented per 1,000 patients.

### Statistical analysis

Failure rates were estimated by survival analysis using Kaplan-Meier method. Recurrence of parasitaemia was treated as failure and patients lost to follow-up were censored at the last follow-up visit.

The association between the area under the gametocyte density time curve (AUC) and gametocyte count on day 7 was examined using Spearman's correlation. AUC was calculated using trapezoid method over 28 day follow-up period and was restricted to patients who had measurements taken on day 0,7,14 and 28. To calculate AUC and AUIC, it was assumed that missing counts which follow gametocytaemia clearance (i.e. at least one negative count after positive counts) remained negative. To avoid confounding with recrudescence or reinfection in assessing the asexual to sexual stage transition, the ratio between maximum gametocytaemia and maximum asexual parasitaemia was calculated only in patients with maximum gametocytaemia recorded within 14 days after starting treatment. Parasitaemia on enrolment was used for the maximum parasitaemia.

For the purpose of analyses the following variables were dichotomized: temperature (oral > = 38C or axillary > 37.5C), history of illness (> = 3 days), liver size (> 0 cm), spleen size (> 0 cm). Anaemia and severe anaemia were defined on the basis of haematocrit using cutoffs of 30% and 20% respectively. Parasitaemia on enrolment was examined as a categorical variable. The effect of continuous parasitaemia (after log 10 transformation) was also examined in studies with available data.

The duration of gametocyte carriage was analysed by survival methods. Kaplan-Meier estimates (K-M) of gametocyte carriage rates were calculated at day 7 and 14 and compared between groups using the log-rank test stratified by study site. Heterogeneity of results between studies within each country was examined through the test of interaction in the Cox proportional hazards model. As the proportional hazards assumption was not satisfied between sites, effects of covariates in the combined dataset and their interaction with site were examined in a lognormal model with accelerated failure-time parameterization and with gamma frailty (to account for study and site effects) [32].

Associations between maximum gametocytaemia and explanatory variables were examined using a negative binomial regression model [33,34] with random effects to accommodate study variation in dispersion. Results were summarized in groups of patients by ratios of mean densities (exponent of the regression coefficients). Only patients who carried gametocytes at some time during the follow-up contributed to this analysis.

Relationships between binary outcomes and explanatory variables were examined using the Mantel-Haenzel method, stratified by study site. The effects of covariates and their interaction with country in the combined data set were explored using logistic regression models with random effects. In all analyses, unless stated otherwise, age, haematocrit and enrolment parasitaemia were treated as continuous covariates. Enrolment parasitaemia was log-transformed and haematocrit was adjusted for each site population median.

In multivariate analyses, all covariates examined in corresponding univariate analyses were investigated. Final models were selected by stepwise forward variable selection procedure and only covariates significant at 5% were included in the final model. For continuous variables categorical and continuous representations were investigated and the one which maximized the log-likelihood function was selected. If the difference in the log-likelihood was not significant, the continuous representation was selected.

All analyses were performed on the maximum possible data set, i.e. on all patients with non-missing required data and since different analysis used different endpoints and covariates different subsets of patients were analysed throughout the paper. Numbers of patients used in each analysis are given.

## Results

In total, there were 10,968 patients with confirmed falciparum malaria on enrolment and at least one gametocyte assessment available on enrolment or during the follow-up. Treatments administered and their efficacies are summarized in Table 1.

**Figure 2:**
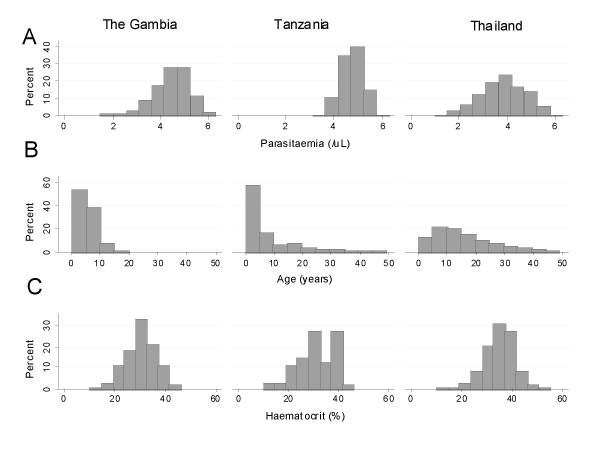
Distribution of admission characteristics of patients in the three countries: (A) parasitaemia, (B) age, (C) haematocrit.

The demographic characteristics of patients in the three populations studied are shown in Table 2. There was a significant difference in the age distribution between sites. After adjustment for age the patients in Thailand were less anaemic (p < 0.001) and had lower parasite counts (p < 0.001) than in the two African study sites. Figure 2 shows the distributions of enrolment parasitaemia, age and haematocrit for the three sites.

**Table 2:** Demographic characteristics of patients in the three populations

	**Thailand**	**The Gambia**	**Tanzania**
**Total number of patients**	7548	2020	1400

**Age (years)^1^**	13 (2–43)	4 (1–10)	4 (1–32)

**Male^2^**	3183 (56%)	613 (54%)	487 (43%)

**History of illness (days)^1^**	2 (0–5)	No Data	3 (0–7)

**Symptoms present^2^**	5458 (97%)	No Data	922 (88%)

**Haematocrit (%)^1^**	35 (25–44)	30 (20–39)	31 (18–40)^4^

**Palpable liver^2^**	868 (16%)	221 (20%)	No Data

**Palpable spleen^2^**	1197 (22%)	517 (45%)	No Data

**Axillary temperature^1^**	37.7 (36.1–39.6)	37.8 (36.2–40)	No Data

**Parasitaemia (/μL)^1^**	6,920 (166–183,920)	32,500 (1,360–311,575)	59,400 (7,992–271,913)^5^

**Parasitaemia (/μL)^2,3^**			
**≤ 1,600**	1,867 (25%)	117 (6%)	395 (28%)
**1,600–4,000**	1,100 (15%)	142 (7%)	454 (32%)
**4,000–20,000**	2,206 (29%)	499 (25%)	262 (19%)
**> 20,000**	2,330 (31%)	1,262 (62%)	289 (21%)

^1 ^median and 90% range are presented.

^2 ^number (%) of patients with this characteristic.

^3 ^n = 49 patients in Thailand had confirmed parasitaemia on admission but the actual count was not recorded.

^4 ^data available for n = 65.

^5 ^data available for n = 257.

### General description

Overall, 19% (2044/10,968) of patients had patent gametocytaemia during the observation period: 12% (931/7548) in Thailand, 34% (683/2020) in The Gambia and 31% (430/11400) in Tanzania (p < 0.001, chi-square test). Approximately one third (33%, 680/2044) of the patients with gametocytaemia at some stage of the infection, already had patent gametocytaemia at enrolment (day 0 or day 1): 35% (318/931) in Thailand, 37% (250/683) in The Gambia, 26% (112/430) in Tanzania. Maximum gametocyte densities were different between the sites, being lowest in Tanzania (median = 55/μL), highest in The Gambia (median = 256/μL) and with a median maximum gametocytaemia of 180/μL in Thailand (p < 0.001, Kruskal-Wallis test). In patients presenting with gametocytes the maximum recorded gametocytaemia occurred on or before day 7 in 99% of Thai patients, 90% of those in Tanzania and 95% in patients from The Gambia. In patients who developed gametocytaemia after enrolment, the maximum gametocytaemia was usually recorded on day 7 but in 30% patients in Gambia, 40% in Tanzania, and 11% in Thailand it occurred after day 7 of follow-up. The area under the gametocyte count-time curve was correlated with gametocytaemia level on day 7; Spearman correlation coefficient of 0.86 (The Gambia; n = 416), 0.57 (Tanzania, n = 198) and 0.42 (Thailand; n = 729) (all p < 0.001). The median (IQR) number of measurements per patients was 4 (4–4), 5(4–5) and 8 (7–11), respectively.

The ratio between maximum gametocytaemia and maximum asexual parasitaemia (a measure of the asexual to sexual stage transition rate) was the lowest (median, 90% range) in Tanzania 0.0008 (0.0001–0.04) n = 16, compared with The Gambia 0.01 (0.0002–1.10) n = 614, and Thailand 0.04 (0.0005–1.77) n = 725 (p < 0.001, Kruskal-Wallis test). The ratio was significantly lower (p < 0.001) in the youngest age group in The Gambia and there was no difference between age groups in Thailand. The ratio was also significantly lower in patients who developed gametocytes after enrolment (median [90% range] = 0.01 [0.0002–0.80]) as compared to those with gametocytes at enrolment (0.05 [0.001–2.67]); p < 0.001. In patients who had any parasitaemia assessments between day 0 and day 7, 79% had maximum parasitaemia recorded on day 0. In the others, the ratio between maximum parasitaemia and enrolment parasitaemia was median (90% range) = 2.6 (1–50).

### Gametocytaemia on enrolment

Overall, 12% (250/2018) of The Gambian patients had gametocytaemia recorded on enrolment compared to 8% (112/1400) in Tanzania and 4% (318/7502) in Thailand (Table 3); p < 0.001.

**Table 3:** Univariate analysis of gametocytaemia at enrolment

	**% (N) with gametocytes at enrolment** **OR [95% CI]** **P-value**			
	
	**All** **(N = 10,920)**	**Thailand** **(N = 7,502)**	**The Gambia** **(N = 2,018)**	**Tanzania** **(N = 1400)**
**Total^1^**	6 (680) < 0.001^1^	4 (318)	12 (250)	8 (112)

**Age (years)^2^**				
**0–5**	8 (270)	2 (31)	13 (151)	11 (88)
**5–15**	5 (203)	3(90)	13 (99)	5 (14)
**> 15**	6 (207)	6 (197)	0 (0)	4 (10)
	0.980 [0.971–0.988]	0.986 [0.975–0.997]	0.962 [0.917–1.009]	0.966 [0.950–0.983]
	< 0.001	< 0.013	0.114	< 0.001

**Symptoms**				
**Yes**	3 (195)	2 (103)	No Data	10 (92)
**No**	5 (17)	4 (7)		8 (10)
	0.962 [0.576–1.608]	0.510 [0.228–1.142]		1.330 [0.673–2.627]
	0.883	0.095		0.410

**History of illness**				
**= 3 days**	6 (64)	4 (28)	No Data	11 (36)
**< 3 days**	3 (147)	2 (79)		8 (68)
	1.659 [1.214–2.267]	1.978 [1.250–3.130]		1.445 [0.943–2.216]
	0.001	0.003		0.089

**Sex**				
**Male**	5 (223)	2 (59)	19 (114)	10 (50)
**Female**	5 (190)	2 (51)	16 (85)	8 (54)
	1.129 [0.918–1.389]	0.912 [0.623–1.336]	1.213 [0.888–1.657]	1.271 [0.848–1.905]
	0.250	0.637	0.225	0.244

**Severe Anaemia**				
**Yes**	33 (36)	17 (7)	43 (27)	40 (2)
**No**	5 (304)	2 (83)	13 (200)	34 (21)
	4.475 [2.856–7.011]^4^	10.331 [4.459–23.462]^4^	4.322 [2.494–7.491]	1.238 [0.189–8.126]
	< 0.001	< 0.001	< 0.001	0.834

**Anaemia**				
**Yes**	14 (204)	6 (47)	21 (144)	50 (13)
**No**	3 (136)	1 (43)	9 (83)	26 (10)
	2.921 [2.305–3.703]^4 ^	4.346 [2.902–6.510]^4^	2.477 [1.830–3. 354]	2.900 [0.970–8.671]
	< 0.001	< 0.001	< 0.001	0.050

**Haematocrit^2^**	0.877 [0.860–0.895]^4^	0.819 [0.789–0.850]^4^	0.897 [0.874–0.920]	0.942 [0.882–1.007]
	< 0.001	< 0.001	< 0.001	0.080

**Palpable liver**				
**Yes**	7 (72)	3 (28)	20 (44)	No Data
**No**	4 (227)	2 (75)	17 (152)	
	1.613 [1.178–2.209]	1.978 [1.269–3.084]	1.355 [0.868–2.116]	
	0.003	0.002	0.179	

**Palpable spleen**				
**Yes**	8 (137)	3 (38)	19 (99)	No Data
**No**	3 (164)	2 (64)	16 (100)	
	1.552 [1.296–1.998]^4^	2.183 [1.464–3.254]^4^	1.288 [0.928–1.786]	
	< 0.001	< 0.001	0.129	

**Axillary Temperature**				
**> = 37.5C**	3 (133)	1 (41)	13 (92)	No Data
**< 37.5C**	7 (176)	3 (68)	24 (107)	
	0.494 [0.404–0.604]	0.660 [0.554–0.786]	0.502 [0.367–0.687]	
	< 0.001	< 0.001	< 0.001	

**Mixed infection on admission**				
**Yes**	-	1 (6)	No Data	No Data
**No**		5 (312)		
		0.354 [0.152–0.821]		
		0.011		

**Parasitaemia^2,3^(/μL)**				
**≤ 1,600**	7 (170)	5 (88)	22(26)	14(56)
**1,600–4,000**	6 (101)	5 (54)	14(20)	6 (27)
**4,000–20,000**	8 (224)	5 (195)	20(100)	7 (19)
**> 20,000**	5 (185)	3 (71)	8(104)	3 (10)
	0.688 [0.632–0.749]^4^	0.517 [0.443–0.604]	0.600 [0.495–0.728]	0.681 [0.543–0.853]
	< 0.001	< 0.001	< 0.001	0.001

^1 ^p-value from chi-square test

^2 ^OR and test for the continuous representation;

^3 ^The Gambia, Thailand: OR calculated for log10 parasitaemia; Tanzania, All : OR for ordered categories, p-value is for test for trend.

^4 ^p-value for homogeneity < 0.05

Risk factors for admission gametocyte carriage included: lower parasitaemia and anaemia in all sites; younger age in Thailand and Tanzania; lower body temperature in Thailand and The Gambia; and longer history of illness, palpable liver, palpable spleen, and pure *P. falciparum *infection in Thailand (Table 3).

**Figure 3:**
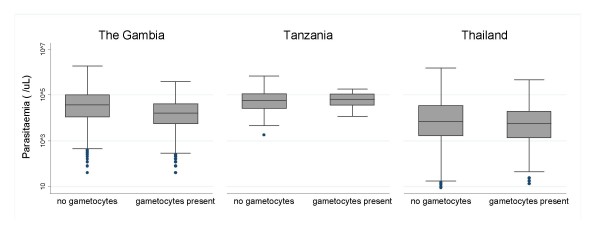
Box plots of admission parasitaemia for patients in the three countries with/without gametocytes present.

**Figure 4:**
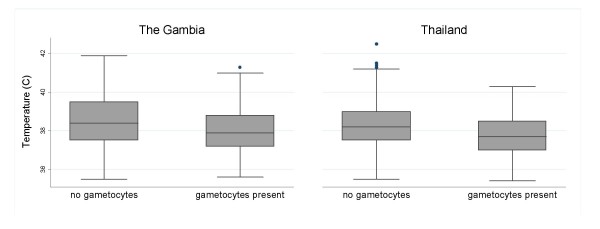
Box plots of admission body temperature for patients in the three countries with/without gametocytes present.

**Figure 5:**
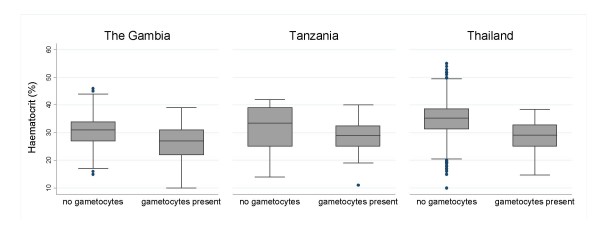
Box plots of admission haematocrit for patients in the three countries with/without gametocytes present.

Figures 3, 4, 5 show the distributions of enrolment parasitaemia, haematocrit and temperature for patients with and without gametocytes at enrolment, by site.

A multivariate analysis could be conducted in three sites separately (Table 4) and for combined data from Thailand and The Gambia only because of different characteristics being collected in Tanzania. For The Gambia, Thailand and their combined data, only enrolment parasitaemia, haematocrit and temperature were selected in the final model. In the combined model, after adjusting for these covariates, gametocyte carriage rates were still much lower in Thailand (OR [95%CI] = 0.048 [0.029–0.075], p < 0.001) then in The Gambia. Although duration of illness and presence of mixed infection was a risk factor in Thailand they could not be tested in the overall model as they were not recorded in The Gambia. The odds ratios confirm the univariate results, and for parasitaemia (OR [95%CI] = 0.580 [0.492–0.683], p < 0.001) and temperature (OR [95%CI] = 0.593 [0.452–0.779], p < 0.001) were similar for the two sites, whereas there were significantly different values for enrolment haematocrit (OR [95%CI] = 0.902 [0.876–0.930] in The Gambia and 0.834 [0.805–0.864] in Thailand, p < 0.001, likelihood ratio test). In Tanzania only age and parasitaemia were selected in the final model.

**Table 4:** Multivariate analysis – gametocytaemia at enrolment.

	**OR [95% CI]** **P-value**		
	
	**Thailand** **(N = 4432)**	**The Gambia^1^** **(N = 1091)**	**Tanzania^1^** **(N = 1383)**
**Log10 Parasitaemia**	0.448 [0.373–0.612]	0.688 [0.549–0.862]	No Data
	< 0.001	0.001	

**Parasitaemia (/μL)**	NS	NS	
**< 1,600**			1.000
**1,600–4,000**			0.266 [0.161–0.440]
			< 0.001
**4,000–20,000**			0.293 [0.164–0.524]
			< 0.001
**≥ 20,000**			0.175 [0.067–0.455]
			< 0.001

**Haematocrit**	0.830 [0.8001–0.861]	0.900 [0.873–0.927]	No Data
	< 0.001	< 0.001	

			No Data
**Axillary Temperature**			
**< 37.5**	1.000	1.000	
**> = 37.5**	0.550 [0.348–0.867]	0.629 [0.447–0.886]	
	< 0.001	0.008	

**Age (years)**	NS	NS	
**0–5**			1.000
**5–15**			0.244 [0.133–0.447] < 0.001
**> 15**			0.155 [0.077–0.311] < 0.001
			

Only covariates significant in at least one model are shown.

^1 ^fixed effects model was fitted since test for random effects not significant.

NS – not significant in the multivariate model.

No Data – no data was collected at the site

### Gametocytaemia after enrolment

In those patients who presented without gametocytes on enrolment, only 3% (122/4,083) overall developed gametocytes carriage during follow up after treatment with artemisinin derivatives. This proportion varies across sites. It was highest in The Gambia; 15% (96/645) compared with 0% (0/100) and 1% (26/3338) in Tanzania and Thailand. However, it should be noted that 10 patients in Tanzania and 109 in Thailand had gametocytaemia recorded between days 1 and 6 but as their readings were negative on day 7 and 14, they were included in these comparisons as not gametocytaemic. Sulphadoxine-pyrimethamine (SP) was associated with the greatest carriage rates (64% 221/348) and this effect was similar in Tanzania and The Gambia (p = 0.215, chi square test). The corresponding overall rates for developing patent gametocytaemia on day 7 or day 14 after the other treatments were 29% (114/390) following chloroquine, 3% (10/349) following halofantrine, 6% (53/878) after mefloquine, and 22% (34/153) after quinine.

In the univariate analyses (Table 5), statistically significant associations were also found between the development of gametocytaemia and young age, long history of illness, anaemia and high parasitaemia on enrolment. In Thailand also palpable spleen, palpable liver and pure *P. falciparum *infection were associated with increased risk of gametocytaemia. In the multivariate analyses (Table 6), in Thailand and The Gambia, low haematocrit on enrolment (OR [95%CI] = 0.875 [0.852–0.898] in Thailand and 0.965 [0.938–0.994] in The Gambia) was associated with an increased risk of the appearance of gametocytaemia. In addition, Thai patients with a palpable spleen (OR [95%CI] = 1.433 [1.050–1.955]) and long history of illness (OR [95%CI] = 2.909 [2.126–3.981]) and pure *P.falciparum *infection (OR [95%CI] = 2.762 [1.559–4.901]) and Gambian patients with high parasitaemia on enrolment (OR [95%CI] = 1.503 [1.177–1.919]) were at increased risk of developing gametocytaemia In all countries, treatment with artemisinin derivatives alone or combined with other drugs resulted in the lowest risk of gametocytaemia. In Tanzania, apart from the treatment, only young age was associated with gametocyte carriage after enrolment. Different sampling schedules prohibited us from combining data from different sites.

**Table 5:** Univariate analysis-presence of gametocytes after enrolment up to day 14

	**% (N) with gametocytes** **OR [95%]** **P-value**			
	
	**All** **(N = 6793)**	**Thailand** **(N = 5069)**	**The Gambia** **(N = 1110)**	**Tanzania** **(N = 614)**
**Total^1^**	15 (998)	7 (342)	36 (400)	42 (256)
	< 0.001^2^			

**Age (years)^3^**				
**0–5**	28 (530)	13 (118)	40 (249)	41(163)
**5–15**	16 (432)	10 (202)	33 (159)	56 (62)
**> 15**	13 (293)	12 (251)	75 (3)	40 (39)
	0.985 [0.979–0.992]	0.987 [0.979–0.995]	0.963 [0.918–1.011]	0.978 [0.963–0.993]
	< 0.001	0.001	0.131	0.005

**Symptoms**				
**Yes**	14 (546)	10 (364)	No Data	51 (182)
**No**	25 (42)	9 (10)		57 (32)
	0.936 [0.586–1.486]	1.147 [0.580–2.268]		0.0.784 [0.444–1.387]
	0.784	0.694		0.403

**History of illness**				
**> = 3 days**	30 (193)	25 (129)	No Data	48 (64)
**< 3 days**	12 (408)	8 (234)		57 (174)
	2.030 [1.658–2.484]^5^	3.400 [2.640–4.380]		0.683 [0.453–1.029]
	< 0.001	< 0.001		0.067

**Sex**				
**Male**	18 (446)	10 (203)	39 (136)	57 (107)
**Female**	19 (421)	10 (172)	37 (117)	52 (132)
	1.071 [0.910–1.261]	1.041 [0.836–1.295]	1.052 [0.766–1.445]	1.201 [0.821–1.758]
	0.406	0.722	0.754	0.345

**Severe Anaemia**				
**Yes**	33 (18)	29 (7)	37 (11)	No Data
**No**	17 (636)	10 (287)	34 (341)	
	1.536 [0.858–2.747]^5^	2.433 [0.989–5.986]^5^	1.302 [0.586–2.893]	
	0.145	0.045	0.515	

**Anaemia**				
**Yes**	32 (294)	26 (136)	38 (157)	No Data
**No**	12 (360)	7 (158)	32 (195)	
	2.308 [1.920–2.775]^5^	4.135 [3.156–5.418]	1.402 [1.069–1.840]	
	< 0.001	< 0.001	0.014	

**Haematocrit^3^**	0.917 [0.903–0.932]^5^	0.872 [0.853–0.891]	0.974 [0.950–0.998]	No Data
	< 0.001	< 0.001	0.037	

**Palpable liver**				
**Yes**	21 (156)	17 (100)	40 (56)	No Data
**No**	13 (449)	9 (256)	37 (193)	
	1.725 [1.378–2.162]	1.965 [1.513–2.551]	1.216 [0.772–1.918]	
	< 0.001	< 0.001	0.398	

**Palpable spleen**				
**Yes**	22 (253)	17 (143)	40 (110)	No Data
**No**	11 (355)	8 (212)	36 (143)	
	1.708 [1.412–2.065]^5^	2.227 [1.756–2.826]^5^	1.067 [0.768–1.483]	
	< 0.001	< 0.001	0.698	

**Temperature**				
**> = 37 C**	14 (385)	9 (213)	40 (172)	No Data
**< 37.5 C**	15 (242)	11 (161)	33 (81)	
	0.947 [0.785–1.141]	0.831 [0.664–1.040]	1.273 [0.904–1.794]	
	0.564	0.106	0.165	

**Parasitaemia^3,4^(/μL)**				
**≤ 1,600**	13 (187)	9(110)	25 (16)	46 (61)
**1,600–4,000**	20 (205)	10(76)	30 (28)	57 (101)
**4,000 – 20,000**	18 (355)	12(185)	34 (100)	55 (70)
**> 20,000**	21 (502)	13 (200)	41 (267)	20 (35)
	1.120 [1.013–1.238]	1.053 [0.936–1.184]	1.335 [1.907–1.625]	1.182 [0.960–1.458]
	0.027	0.394	0.004	0.115

**Mixed infection**				
**Yes**		4 (22)		
**No**	No Data	12 (549)	No Data	No Data
		0.366 [0.236–0.567]^5^		
		< 0.001		

**Treatment^6^**				
**A**	10 (413)	8 (287)	16 (106)	10 (11)
**C-F**	26 (516)	18 (276)	27 (13)	49 (227)
	3.073 [2.384–3.962]	3.063 [2.330–4.026]	3.259 [1.028–10.387]	4.751 [1.530–14.754]
	< 0.001	< 0.001	0.033	0.003
**G-H**	70 (301)	No Data	71 (283)	62 (18)^7^
	11.744 [8.019–17.199]		11.936 [8.136–17.510]	
	< 0.001		< 0.001	

^1 ^Percentages are calculated from patients with both non-missing counts on day 7 and day 14 (out of 4840, 1099 and 603 patients, respectively)

^2 ^p-value from chi-square test

^3 ^OR and test for the continuous representation

^4 ^The Gambia, Thailand: OR calculated for log10 parasitaemia; Tanzania, All : OR for ordered categories, p-value is for test for trend.

^5 ^p-value for homogeneity < 0.05

^6 ^treatments groups are defined as: A: artemisisnin combination alone or with other treatments; C-F: other monotherapies (see Table 1); G-H: SP alone or SP with chloroquine. OR are calculated with reference to group A.

^7 ^G-H/A at separate sites

**Table 6:** Multivariate analysis for gametocytaemia after enrolment up to day 14.

	**OR [95% CI]** **P-value**		
	
	**Thailand** **(N = 2633)**	**The Gambia** **(N = 1031)^1^**	**Tanzania** **(N = 592)**
**Log 10 parasitaemia**	NS	1.503 [1.177–1.919] 0.001	No Data

**History of illness**		No Data	NS
**< 3 days**	1.000		
**> = 3 days**	2.909 [2.126–3.981]		
	< 0.001		

**Haematocrit**	0.875 [0.852–0.898]	0.965 [0.938–0.994]	No Data
	< 0.001	0.017	

**Palpable spleen**		No Data	NS
**No**	1.000		
**Yes**	1.433 [1.050–1.955]		
	0.023		

**Mixed infection**	0.362 [0.204–0.641]	No Data	No Data
	0.001		

**Treatment^2^**			
**A**	1.000	1.000	1.000
**C-F**	2.964 [2.048 4.290]	1.661 [0.709–3.893]	3.432 [1.483–7.946]
	< 0.001	0.243	0.004
**G-H**		12.320 [8.757–17.333]	5.520 [1.724–17.675]
		< 0.001	0.002

**Age (years)**	NS	NS	
**0–5**			1.000
**5–15**			0.750 [0.465–1.210] 0.238
**> 15**			0.409 [0.250–0.671] < 0.001

Only covariates significant in at least one model are shown.

^1 ^fixed effects model was fitted since test for random effects not significant

^2 ^treatments groups are defined as: A: artemisisnin combination alone or with other treatments;

C-F: other monotherapies (see Table 1); G-H: SP alone or SP with chloroquine. OR are calculated with reference to group A.

### Length of gametocyte carriage

Of the 2027 patients with gametocytes before day 28, 12 (0.6%) had three and 164 (8%) had two separated episodes of gametocytes carriage, with a negative count between positive counts. Among a total of 2213 episodes, only 58 (3%) had one missing measurement and 7 (0.3%) episodes had 2 or more missing measurements between the first and last counts. Quality of the follow-up information varied between sites, with 68% censored observations in The Gambia, 61% in Tanzania and 19% in Thailand, although one third of these in The Gambia and one quarter in Tanzania were censored after day 14. The proportion of patients who had missing measurements before the first positive gametocyte count was 7%, 6% and 2% in Tanzania, The Gambia and Thailand.

Gametocyte carriage rates (95% CI) after 7 and 14 days were smallest in Thailand (Table 7): 34(31–37)% and 19(16–22)%. In The Gambia and Tanzania rates were very similar (p = 0.99, logrank test) and at least twice the rates in Thailand. In each site, gametocyte carriage rates after 7 days in patients who were treated with artemisinin derivatives alone or with other drugs were significantly lower than rates of carriage in patients on other treatments: 33(30 – 36)% versus 62% (58–65)% for patients treated with monotherapies (groups C-F) and 85 (71–88)% for patients treated with sulphadoxine-pyrimethamine (groups G-H) in all sites combined (p < 0.001, logrank test stratified by study). The corresponding gametocyte carriage rates (95%CI) after 14 days were 18 (15–21)%, 47 (43–51)% and 80 (75–84)%.

**Table 7:** Estimated gametocyte carriage rates (GCR)

	**All** **(N = 2027)**	**Thailand** **(N = 922)**	**The Gambia** **(N = 681)**	**Tanzania** **(N = 424)**
**GCR (%) after 7 days (95% CI)**				

**Overall**	54 (52–56)	34 (31–37)	65 (61–69)	71 (66 – 75)

**Gametocytaemia on enrolment**				
**No**	53 (50–55)	32(28–36)	71 (66–75)	69 (64–75)
**Yes**	58 (39–45)	40 (35–45)	77 (71–82)	75 (66–82)

**Treatment**				
**A**	33 (30–36)	24(20–28)	54 (48–60)	26 (5–53)
**C-F**	62 (58–65)	50 (44–55)	81 (63–91)	73 (68–78)
**G-H**	85 (71–88)		86 (82–89)	66 (45–80)

**GCR (%) after 14 days (95% CI)**				

**Overall**	41 (38–43)	19 (16–22)	65 (61–69)	57 (51–63)

**Gametocytaemia on enrolment**				
**No**	42 (39–45)	18 (14–21)	71(66–75)	53(46–60)
**Yes**	41(37–45)	23 (18–28)	58 (51–64)	65 (54–74)

**Treatment^1^**				
**A**	18(15–21)	9 (7–12)	38 (32–45)	13(1–42)
**C-F**	47(43–51)	34 (29–40)	81 (63–91)	60 (54–66)
**G-H**	80 (75–84)		83 (77–87)	38 (18–58)

^1 ^treatments groups are defined as: A: artemisisnin combination alone or with other treatments;

C-F: other monotherapies (see Table 1); G-H: SP alone or SP with chloroquine.

In Thailand other risk factors for prolonged carriage included high enrolment parasitaemia (p = 0.005), a prolonged history of illness (p = 0.038) and anaemia (p = 0.034). These associations were not apparent in the other sites.

Site, presence of gametocytaemia on enrolment, haematocrit and treatment were all independent predictors of the duration of gametocyte carriage in the multivariate analysis of the combined data from Thailand and The Gambia (Table 8). In Thailand carriage was 86% shorter than in The Gambia. In patients who received artemisinin derivatives carriage was shortened in duration by 27% in Thailand and by 71% in the Gambia. In The Gambia, gametocytaemia present before treatment had a 72% longer clearance time than gametocytaemia which appeared after treatment. In Thailand the difference in clearance time was not significant. Carriage was longer in people presenting with anaemia by 27% in both countries.

**Table 8:** Parameter estimates from the exponential model with gamma frailty for the length of gametocytes carriage, data from Thailand and The Gambia combined, N = 971

	**Exp(Coefficient)^1^**	**95% CI**	**P-value**
**Study Site**			
**Thailand**	0.139	0.099–0.193	< 0.001
**The Gambia**	1.000		

**Anaemia**	1.266	1.071–1.496	0.006

**Treatment with artemisinin derivatives**			
**Thailand**	0.725	0.549–0.958	0.024
**The Gambia**	0.293	0.226–0.380	< 0.001

**Presence of gametocytes before treatment**			
**Thailand**	0.934	0.704–1.239	0.636
**The Gambia**	1.723	1.335–2.222	< 0.001

^1 ^Represents change in length of gametocytes carriage relative to the rest of the population

When a common model was fitted for all three data sets using common covariates (anaemia could not be included since it was missing for majority of patients in Tanzania), gametocyte carriage in Tanzania was estimated to be shorter by 65% than gametocyte carriage in The Gambia. Effects of gametocytaemia on admission and treatment with artemisinin derivatives in Tanzania were estimated to be the same as in The Gambia.

### Maximum gametocytaemia

In Thailand, the maximum gametocyte density was increased in patients with gametocytaemia on enrolment (Incidence Rate Ratio (IRR) [95%CI] = 1.261 [1.081–1.430], p = 0.001, n = 925), severe anaemia (IRR [95%CI] = 1.744 [1.136–2.677], p = 0.011, n = 403) and a prolonged history of illness (> 3 days) (IRR [95%CI] = 1.234 [1.047–1.455], p = 0.019, n = 491). The two children age groups (< 5 years and 5–15 years) had similar levels of maximum gametocytaemia which were significantly higher than in adults (IRR [95%CI] = 1.135 [1.002–1.284], p = 0.046, n = 931). The effects of these covariates were the same for patients presenting with gametocytaemia on enrolment and patients who developed gametocytaemia later, as tested by the interaction term in the model. No covariates were significant in the multivariate analysis.

In The Gambia, the only determinant of maximum gametocyte density was drug treatment, but this effect was different for patients presenting with gametocytaemia on enrolment and patients who developed gametocytaemia later (p = 0.009, n = 651). In both cases, patients treated with SP produced higher number of gametocytes than patients treated with artemisinin derivatives (IRR [95%CI] = 1.880 [1.533–2.306] for patients presenting without gametocytaemia, 1.404 [1.106–1.7] for patients presenting with gametocytaemia).

In Tanzania, higher gametocyte densities were found in patients with high parasitaemia on enrolment (IRR [95%CI] = 1.461 [1.301–1.640] for increase in parasitaemia up to the next category, p < 0.001 test for trend, n = 430), females (IRR [95%CI] = 1.595 [1.244–2.045], p < 0.001, n = 391), anaemic patients (IRR [95%CI] = 2.427 [1.128–5.222], p = 0.012, n = 33), and symptomatic patients (IRR [95%CI] = 1.730 [1.186–2.524], p = 0.004, n = 362). Children aged 5–15 years (IRR [95%CI] = 0.584 [0.431–0.790], p < 0.001) and adults (IRR [95%CI] = 0.253 [0.176–0.363], p < 0.001) had significantly lower gametocyte densities compared to children less than 5 years of age (n = 427). In the multivariate model only age (IRR [95%CI] = 0.583 [0.439–0.795] for 5–15 years, 0.317 [0.207–0.424] for > 15 years, as compared to children 0–5 years) and parasitaemia (IRR [95%CI] = 2.201 [1.542–3.142] for parasitaemia increase up to the next category, p < 0.001, test for trend) remained independent determinants of gametocyte density. Anaemia was not investigated in the multivariate analysis due to the large number of missing values. Since multivariate analyses in each site resulted in models with no common covariates no analysis was performed on the combined data set.

### Predictors of potential infectivity

**Figure 6:**
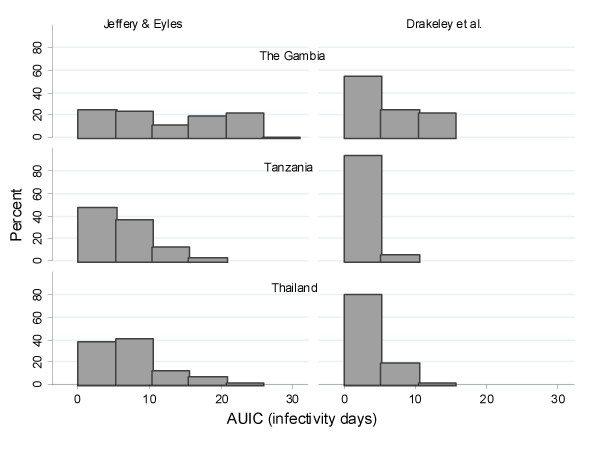
**Distribution of AUIC for patients in the three countries.** Only patients with gametocytes are presented: in The Gambia 426 out of 779 (53%), in Tanzania 170 out of 347 (50%) and in Thailand 421 out of 4064 (10%). Patients who had any missing gametocytes measurements on day 0, 7, 14, 28 were not included unless the missing values occurred after the day of gametocyte clearance.

**Figure 7:**
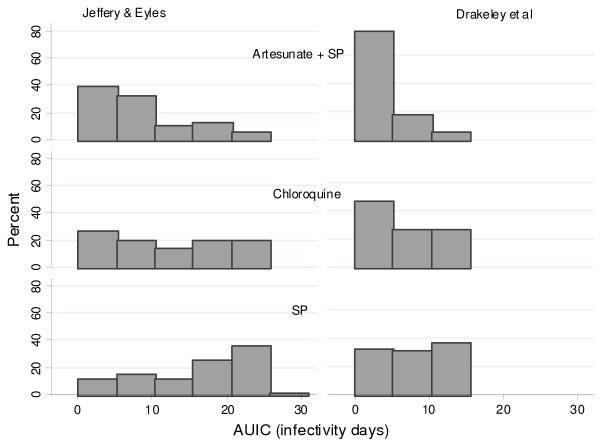
**Distribution of AUIC for patients in The Gambia for different treatment groups.** Only patients with gametocytes are presented: in Artesunate +SP group 197 out of 474 (42%), in SP group 214 out of 289 (74%) and in Chloroquine group 15 out of 36 (42%) patients. Patients who had any missing gametocytes measurements on day 0, 7, 14, 28 were not included unless the missing values occurred after the day of gametocyte clearance.

**Figure 8:**
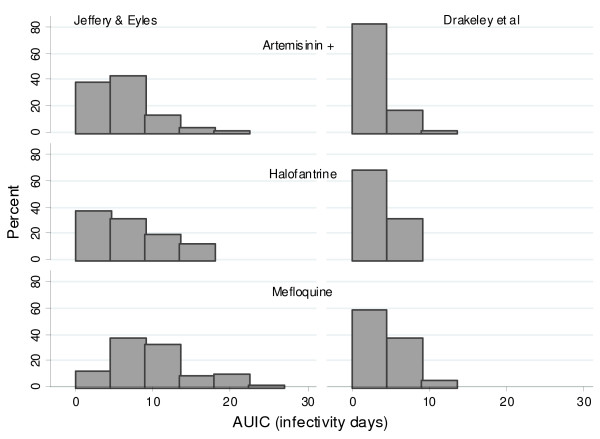
**Distribution of AUIC in patients with gametocytes in Thailand in different treatment groups.** Only patients with gametocytes are presented: 253 patients out of 2864 (9%) in the Artemisinin +Other group, 16 out of 298 (5%) in the Halofantrine group and 113 out of 775 (15%) patients in the Mefloquine group. Patients who had any missing gametocytes measurements on day 0, 7, 14, 28 were not included unless the missing values occurred after the day of gametocyte clearance.

The putative patient infectivity was estimated from the serial gametocyte counts. Among patients with gametocytes measurements available on days 0, 7, 14 and 28, all four blood film examinations were negative for gametocytes in 92% (3,643/3,970) of Thai patients, compared to 63% (177/281) of Tanzanian patients and 54% (373/685) of patients from the Gambia; p < 0.001. These patients were excluded from the analysis of infectivity. In nearly all cases (1012/1017) the area under the infectivity curve (AUIC) calculated using Method A [15] was median (range) 102 (4 – 505)% higher than that calculated using Method B [16]. Among those who carried gametocytes at some stage of the infection, the area under the infectivity curve (median [90% range]) was highest in The Gambia (Method A: 11.2 [2.0–24.2] infectivity days and Method B: 4.4 [1.9–12.04] infectivity days, n = 426), markedly lower in Thailand (Method A: 6.3 [2.6–18.3] and Method B: 2.9 [1.9–11.6] infectivity days, n = 421) and lowest in Tanzania (Method A: 5.6 [2.3–14.3] and Method B: 1.9 [1.9–5.6] infectivity days, n = 170) (Figure 6). Figures 7 and 8 show distribution of AUIC by treatment group in Thailand and The Gambia. In Tanzania majority of patients (78%) received the same treatment. Assuming that Method A is more appropriate for non-immune patients in Thailand while Method B is more appropriate for patients in The Gambia and Tanzania and that 8% of symptomatic patients in Thailand, 37% in Tanzania and 46% in The Gambia will carry gametocytes at some stage of the infection; the estimated overall infectivity of a population of 1000 patients during the 28 days follow-up are 609 person infectivity days in Thailand, 901 in Tanzania and 2688 in The Gambia. Assuming that everybody in this population received one treatment, then these estimates become 486 person infectivity days for artemisinin derivatives, 530 for halofantrine and 771 for mefloquine in Thailand; 1812 person infectivity days for SP+AS and 3474 for SP alone in The Gambia; 892 person infectivity days for chloroquine in Tanzania. The proportion of patients presenting with gametocytes at enrolment was the same for the two treatments in Thailand (5%) and The Gambia (12%).

## Discussion

This comparison between three malariaous regions with very different epidemiological characteristics detected considerable heterogeneity in *P. falciparum *gametocyte carriage. Some of this heterogeneity is explained by known factors such as the differences in age (a proxy for cumulative exposure and thus immunity), levels of anaemia, and type of anti-malarial drug used [14]. On enrolment, before drug treatment, gametocyte carriage in Thailand was 4%. This was three-fold lower than in The Gambia, and two-fold lower than in Tanzania. Within 14 days from starting treatment a further 40% of patients developed gametocytes in The Gambia and Tanzania, while only 7% more patients developed gametocytaemia in Thailand. Duration of carriage was also significantly longer in the African countries than in Thailand. In contrast, gametocytes densities were lowest in Tanzania. All this is reflected in the AUIC estimates with The Gambia having the largest individual estimates and Tanzania the smallest. Nevertheless, after adjusting for the number of gametocytes carriers, even if different relationships appropriate to the immunity status in the three regions were applied (i.e. Method A in Thailand and Method B in The Gambia and Tanzania), the Thai population of symptomatic patients was still the least infectious, with Tanzanian patients being 50% more infectious and patients in The Gambia being nearly four times as infectious.

Admission gametocytaemia was associated independently with lower haematocrit, lower parasitaemia, and lower temperature. Slowing the expansion of asexual parasitaemia may have less effect on the persistence of gametocytaemia, as gametocytes have greater longevity than the asexual stages. In Thailand, where duration of illness was recorded, gametocyte prevalence was associated with a longer period of illness before presentation. In low transmission settings the majority of gametocytaemic individuals will be symptomatic and seek treatment during the acute phase of the illness, and the majority of gametocyte carriage will occur after starting treatment. In the high transmission settings contribution of asymptomatic carriers is significant and it has been demonstrated that carriers with submicroscopic densities of gametocytes are capable of infecting mosquitoes [1,9]. The dynamics of infection in a high transmission setting where multiple infections coexist within the human host may also be more conducive to the production of gametocytes [35]. Whichever the explanation effective anti-malarial treatment will have a much greater impact on overall malaria transmission in areas of low transmission intensity than in high transmission areas, where a smaller fraction of parasitaemic individuals seeks treatment.

However, transmission intensity often varies greatly over small geographic distances, and low transmission settings often contain microfoci of much higher transmission intensity. For example even in Thailand, there is evidence of an untreated reservoir of infectious individuals in some areas [36], although these are now few. Given marked heterogeneity in gametocyte dynamics among sites, it is remarkable that several parameters were consistently associated with gametocyte carriage in all three sites; all of which are indicative of a longer period of infection, which could reflect an effective host response and the development of premunition, poor access to effective treatment in impoverished rural areas, exacerbated by drug resistance or a combination of these [37]. Further, the substantial impact of ACT in reducing post-treatment gametocyte carriage was seen across all studies in all three countries [14].

In nearly all patients with gametocytaemia, densities peaked in the first week after starting treatment (day 0–7). The day 7 gametocyte density proved a good surrogate for the area under the gametocytaemia-time curve. The factors associated with post-treatment gametocytaemia were similar to those associated with enrolment gametocytaemia. There were large differences between treatment regimens; those containing an artemisinin derivative were associated with lower and shorter carriage, and treatment with SP being associated with higher carriage rates and longer duration. Although there is a relationship between the administration of anti-malarial drugs and gametocyte density, these data cannot be translated directly into transmission potential because of the different effects of the drugs on gametocyte viability [1,16,38-40].

As drug resistant parasites become more prevalent, the duration of malaria infections lengthens and the proportion of recrudescent infections increases. Recrudescent infections are cumulatively of longer duration than primary infections, and are associated with higher rates of anaemia and gametocyte carriage [34]. Drug resistant parasites have been associated with higher gametocyte carriage and enhanced mosquito infectivity in the absence of appreciable clinical or parasitological treatment failure [41-43]. Therefore, in high transmission areas, where the impact of treatment on overall malaria transmission is likely to be less than in areas of low endemicity, ACT can provide substantial benefit in reducing the spread of drug resistant parasite genotypes [39].

This study has several limitations. Data sets from several studies were combined for the purpose of this analysis. This was a retrospective analysis so inclusion criteria for the trials were not standardized which resulted in the differences in the populations participating in the three sites. For example, in The Gambia only children were enrolled while in the other two sites a broader age spectrum was enrolled. Different covariates were assessed and laboratory methods were not standardized between the sites. Different detection limits for gametocytes affected the estimated densities and rates of carriage. It is also possible that the apparent differences in gametocyte carriage rates, which are dependent on the frequency of observation, are influenced by these differences in trial conduct. Gametocytes counts were collected at slightly different sampling schedules and there was a considerable number of missing values (20–40%) at visits scheduled for all three sites. The estimates of infectivity are illustrative, being based on two data-sets which characterized the sigmoid relationship between gametocyte densities and infectivity to an anopheline vector. This hides considerable inter-individual variability and the confounding effects of immunity and other factors. Much more data are needed to characterize and quantify the sources of variance in this assessment and produce more valid assessments necessary to model the impact of interventions on malaria transmission.

## Conclusion

This study confirms the independent association of increased rates of gametocyte carriage with anaemia [29,44], and with treatment with sulphadoxine-pyrimethamine [45,46]. Gametocyte carriage is significantly reduced by combination treatment with the artemisinin derivatives (ACT), which were associated with the lowest rates of gametocyte carriage [11,29]. The large differences in gametocyte carriage rates between regions with different levels of malaria transmission and access to treatment suggest that drug interventions to prevent transmission will have different effects in different places. In areas of high stable transmission, less reduction in transmission can be expected from effective treatment, with the major benefit of ACT deployment being reducing the spread of resistance to non-artemisinin drugs. At low levels of transmission, prompt treatment with gametocytocidal drugs can have a major effect on the transmission and thus incidence of falciparum malaria.

## Abbreviations

ACT: Artemisinin combination therapy; AUIC: Area under the infectivity curve; AUC: Area under the gametocyte density time curve; EIR: Entomological inoculation rate; GCR: Gametocyte carriage rate; IQR: Inter-quartile range; IRR: Incidence Rate Ratio; K-M: Kaplan-Meier; OR: Odds ratio; CI: Confidence interval; *P. falciparum*: *Plasmodium falciparum*; *P. malariae*: *Plasmodium malariae*; *P. ovale*: *Plasmodium ovale*; *P. vivax*: *Plasmodium vivax*; SP: Sulphadoxine-pyrimethamine.

## Competing interests

The authors declare that they have no competing interests.

## Authors' contributions

KS analysed the data and drafted the manuscript; RNP, CJS, CJD, LS, FN provided the data and contributed to the manuscript writing, NJW contributed to the manuscript writing and conceptually to data analysis.
